# Supplementary material for: High-throughput, organ-scale 3D tubule tracking using TubuleMAP
Source: Res Sq. 2026 Apr 30:rs.3.rs-9510358. Preprint. [Version 1] doi: 10.21203/rs.3.rs-9510358/v1 (PMC13142624; doi:10.21203/rs.3.rs-9510358/v1)
Supplement: 1 — Supplementary Figure S1: Segmentation performance varies with spatial orientation of tubules. Supplementary Figure S2: TubuleMAP modules. Supplementary Figure S3: Screenshot of the TubuleMAP interface and associated features. Supplementary Figure S4: Characterization of TubuleMAP performance, speed, and scalability. Supplementary Figure S5: Comparing 3D segmentation quality between a manual approach versus TubuleMAP for two nephrons. Supplementary Figure S6: nnU-Net based segmentation of kidney tissue regions. Supplementary Figure S7: Quantification of mesoscale organization of the kidney. Supplementary Figure S8: Characterizing the presence of thin limb or thick limb at the loop of Henle tip. Supplementary Figure S9: Nephrons with atypical morphologies. Supplementary Figure S10: Analysis of spermatogenic waves in seminiferous tubules. Supplementary Figure S11: Screenshots of supplementary videos. Supplementary Video 1: Surface rendering and virtual slices from kidney slab to cropped data with segmented nephron. Supplementary Video 2: TubuleMAP tracking and segmentation strategy. Supplementary Video 3: Parallel processing of tubules trajectories. Supplementary Video 4: TubuleMAP graphical user interface and tracking workflow. Interface is based on napari with multiple widgets for data exploration and human intervention. Supplementary Video 5: Three-dimensional reconstruction of 1000 nephrons from volumetric imaging data. Supplementary Video 6: Spatial distribution of short-loop nephrons and long-loop nephrons across the kidney. Supplementary Video 7: Nuclei distribution in short-loop and long-loop nephrons. Supplementary Video 8: Three-dimensional visualization of seminiferous tubules in cleared testis tissue. Supplementary Video 9: Top: straightened view of the tubule and assignment of spermatogenic wave states. Left bottom: Three-dimensional view of a mouse seminiferous tubule during tracking showing the centerline and orthogonal cross-sections with sampled planes [file NIHPPRS9510358V1-supplement-1.pdf]

SUPPLEMENTARY FIGURES

Supplementary Figure S1: Segmentation performance varies with spatial orientation of tubules.

Supplementary Figure S2: TubuleMAP modules.

Supplementary Figure S3: Screenshot of the TubuleMAP interface and associated features.

Supplementary Figure S4: Characterization of TubuleMAP performance, speed, and scalability.

Supplementary Figure S5: Comparing 3D segmentation quality between a manual approach versus TubuleMAP for two nephrons.

Supplementary Figure S6: nnU-Net based segmentation of kidney tissue regions.

Supplementary Figure S7: Quantification of mesoscale organization of the kidney.

Supplementary Figure S8: Characterizing the presence of thin limb or thick limb at the loop of Henle tip.

Supplementary Figure S9: Nephrons with atypical morphologies.

Supplementary Figure S10: Analysis of spermatogenic waves in seminiferous tubules.

Supplementary Figure S11: Screenshots of supplementary videos.

Supplementary Figure S1

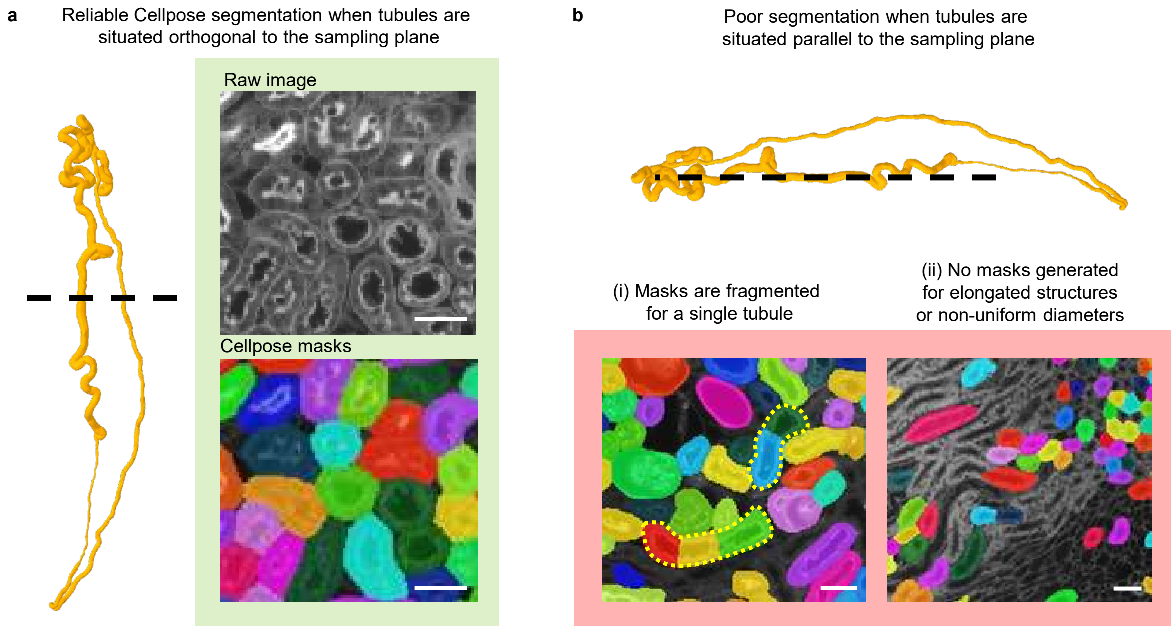

Supplementary Figure S 1: Segmentation performance varies with spatial orientation of tubules. (**a**) Cellpose segmentation shows reliable performance when tubules are situated orthogonal to the sampling plane, cross-sections are mostly circular, and diameters are uniform. (**b**) Cellpose segmentation is poor when tubules are situated parallel to the sampling plane, yielding elongated cross-sections. (i) A single tubule cross-section generates several fragmented segmentation masks (e.g. yellow dashed lines) that need manual joining/correcting, or (ii) most tubule cross-sections do not generate a mask due to elongated structures or different diameters.

Supplementary Figure S2

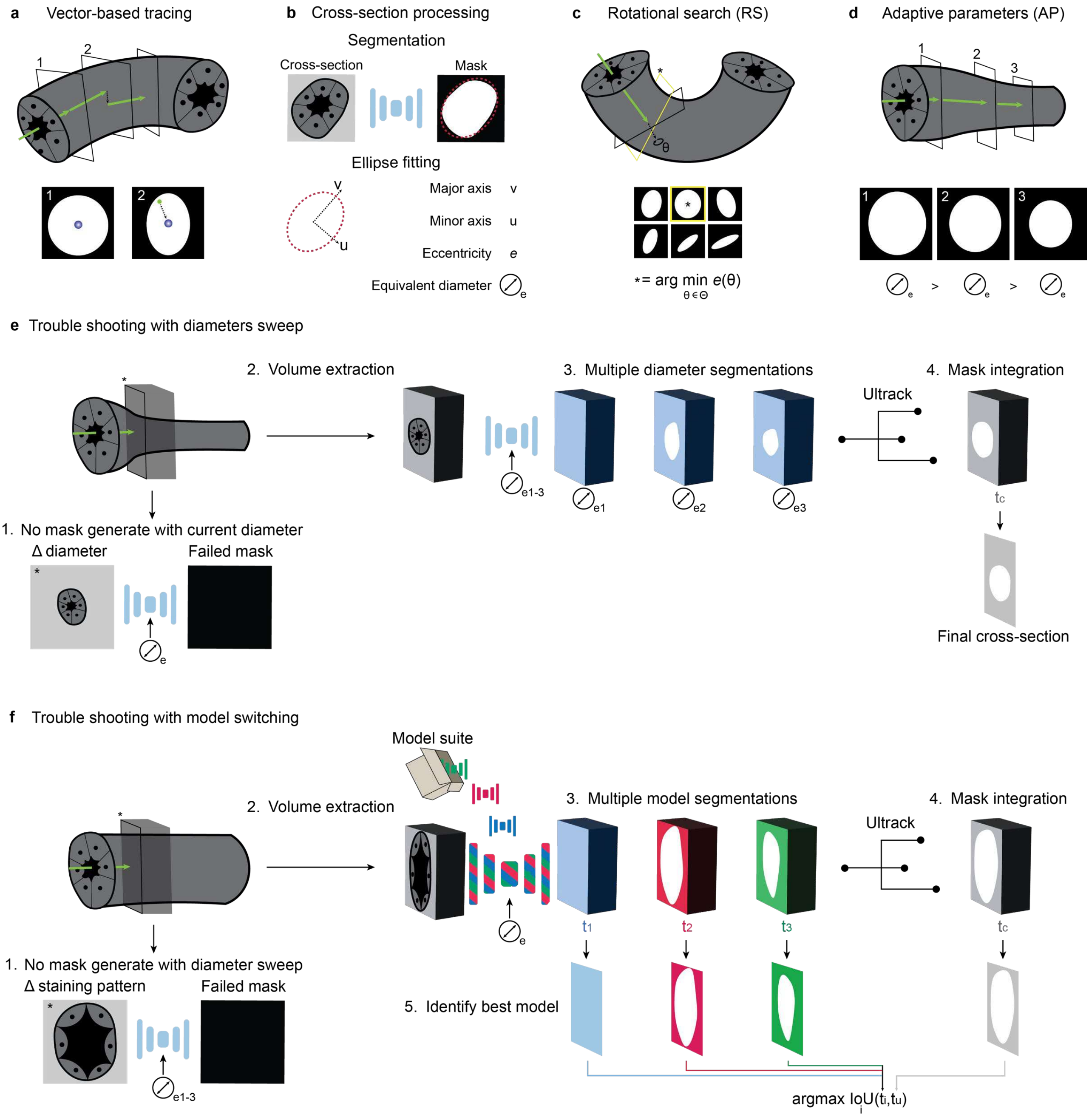

Supplementary Figure S2: TubuleMAP modules. (**a**) Cross-sections obtained at each step are segmented to obtain a mask, which is fit to an ellipse shown by the red dashed outline to quantify its centroid, eccentricity, and equivalent diameter. (**b**) The tracking vector from the previous step is first used to predict the direction for the next step, but is adjusted after segmentation towards the mask centroid. (**c**) At tubule bends where the mask eccentricity can exceed a set threshold, a rotational search is invoked to select the direction with the minimum eccentricity (most circular cross section). (**d**) The equivalent diameter obtained from the latest mask is used to update parameters and adapt to tubule size changes. (**e**) If segmentation fails, a diameter sweep is performed on a local volume sampled along the vector and an optimal diameter is chosen by maximizing overlap with the aggregated mask set. (**f**) If segmentation fails on a diameter sweep, all available segmentation models are applied on the same sampled volume and an optimal model is selected by the same overlap metric. For future steps, we revert to the simpler vector-based tracking using the new optimal model and additional modules are invoked as needed.

Supplementary Figure S3

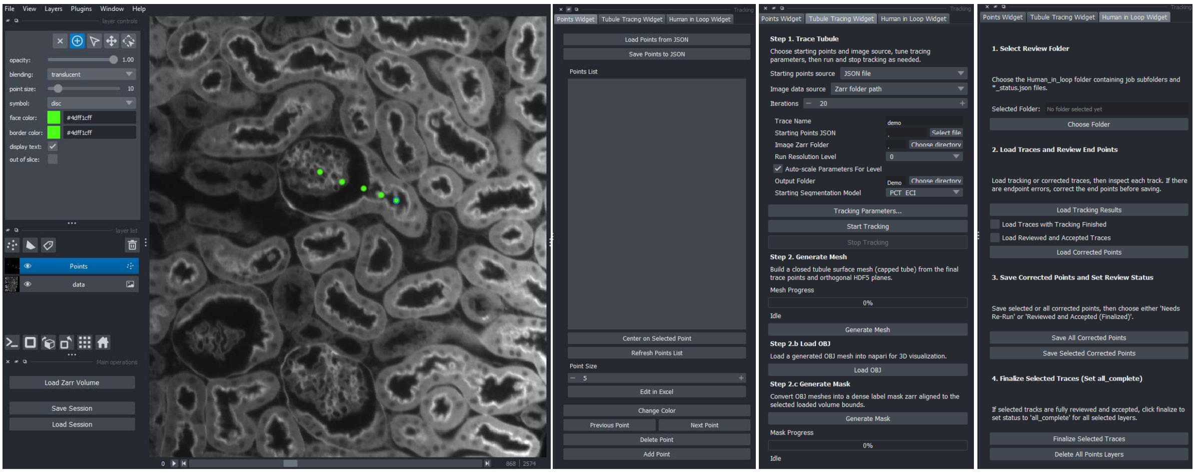

(a) Layers and data input **(b) Napari viewer** **(c) Points widget** **(d) Tracking, mesh and voxellization widget** **(e) Human-in-the-loop widget**

Supplementary Figure S3: Screenshot of the TubuleMAP interface and associated features. (**a**) Widget for layers and data input allows for loading zarr and tif files and creating new points/annotation layers. (**b**) The napari viewer displays the image and provides an interface for human intervention. (**c**) The points widget enables centering of the image on a selected point, toggling between previous and next points, adding or deleting points, and changing colors of individual tracks for visualization. (**d**) The tracking widget lets a user choose various tracking parameters to perform a single tracking run on the GUI itself. The output trajectory is displayed immediately on the napari viewer after each run. 3D meshes based on the segmentation can be generated and loaded directly here, and they can also be voxellized to generate full 3D masks. For high-throughput tracking, a separate multiprocessing script is used and many output tracks can be displayed simultaneously on the GUI. (**e**) The human-in-the-loop widget allows for batch loading and saving tracks with specified status (‘running’, ‘needs correction’, ‘corrected’, or ‘complete’) to quickly review and to organize many files.

Supplementary Figure S4

a Number of human interventions required per nephron segment

(i) TubuleMAP with cellpose ‘cyto2’

Module: TS	Module: RS	Module: AP	Segment: PCT	Segment: Thin limb	Segment: Thick limb
0	0	0	9.8	27.6	48.3
0	0	1	7.2	7.8	5.0
0	1	0	7.5	11.6	0.9
0	1	1	6.2	7.4	6.4
1	0	0	4.3	1.9	8.8
1	0	1	2.8	1.7	6.3
1	1	0	4.6	1.4	8.7
1	1	1	3.3	1.2	7.0

(ii) TubuleMAP with fine-tuned models

Module: TS	Module: RS	Module: AP	Segment: PCT	Segment: Thin limb	Segment: Thick limb
1	0	0	1.7	5.7	2.6
1	0	1	0.7	1.0	1.0
1	1	0	2.4	2.6	4.9
1	1	1	0.6	0.8	1.1

Number of human interventions (colour scale: 50 – 0.5)

b Mean uninterrupted length tracked computationally per nephron segment

(i) TubuleMAP with cellpose ‘cyto2’

Module: TS	Module: RS	Module: AP	Segment: PCT	Segment: Thin limb	Segment: Thick limb
0	0	0	0.3	0.0	0.0
0	0	1	0.5	0.1	0.2
0	1	0	0.5	0.0	0.3
0	1	1	0.6	0.1	0.2
1	0	0	0.8	0.1	0.2
1	0	1	1.1	0.2	0.3
1	1	0	0.7	0.1	0.2
1	1	1	0.9	0.2	0.3

(ii) TubuleMAP with fine-tuned models

Module: TS	Module: RS	Module: AP	Segment: PCT	Segment: Thin limb	Segment: Thick limb
1	0	0	1.7	0.1	0.8
1	0	1	2.4	0.3	1.4
1	1	0	1.4	0.1	0.4
1	1	1	2.6	0.3	1.4

Uninterrupted length tracked (mm) (colour scale: 0 – 3)

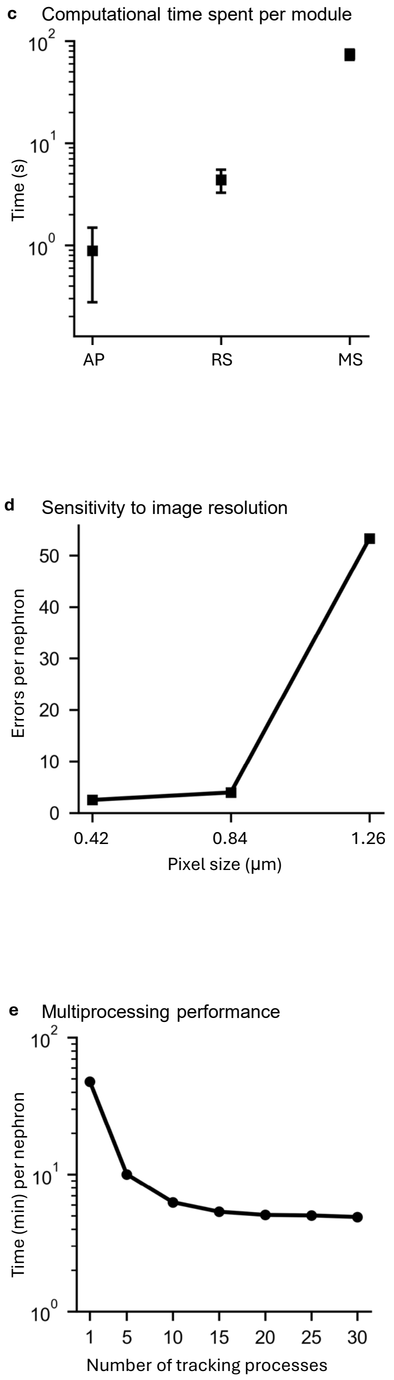

Supplementary Figure S4: Characterization of TubuleMAP performance, speed, and scalability. (**a**) Ablation studies characterizing the number of human interventions required for generalist (Cellpose ‘cyto2’) vs fine-tuned models for tracking different nephron segments after inclusion of various TubuleMAP modules. MS: model switching, RS: rotational search, AP: Adaptive parameters, PCT: proximal convoluted tubule. (**b**) Mean uninterrupted tracked length per nephron segment across TubuleMAP configurations for generalist and fine-tuned models. (**c**) Speed: compute time per step for each TubuleMAP module. (**d**) Resolution sensitivity: impact of spatial resolution on tracking performance. (**e**) Scalability: reduction in tracking time per nephron after parallel processing of tracks on a single NVIDIA RTX 4090 GPU.

Supplementary Figure S5

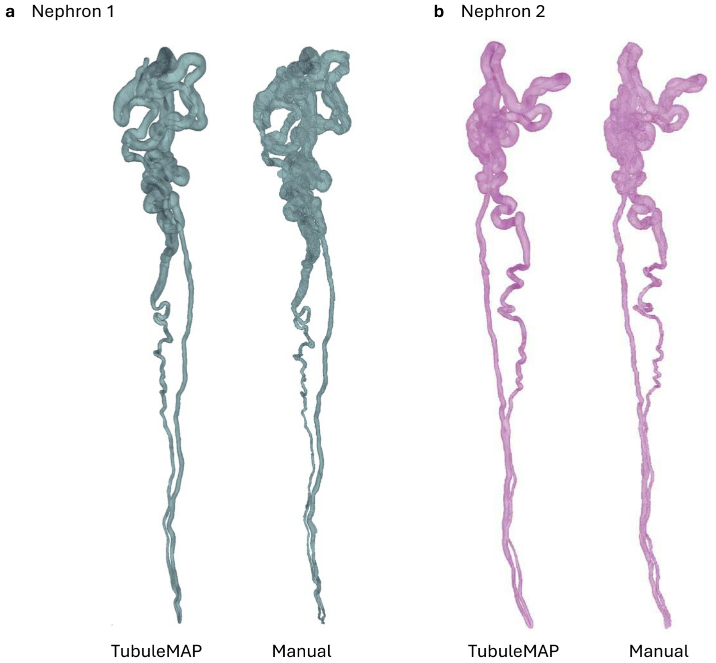

c Performance metrics

Metric	Nephron 1	Nephron 2
IoU	0.73	0.75
F1 score	0.84	0.85
Precision	0.79	0.82
Recall	0.91	0.89

Supplementary Figure S 5: Comparing 3D segmentation quality between a manual approach versus TubuleMAP for two nephrons. (**a-b**) 3D mesh reconstructions of two nephrons generated by TubuleMAP and manual annotation. Nephron 1 is shown in (**a**) and Nephron to shown in (**b**). (**c**) Quantitative performance metrics for each nephron, including Intersection over Union (IoU), Dice score, precision, and recall.

Supplementary Figure S6

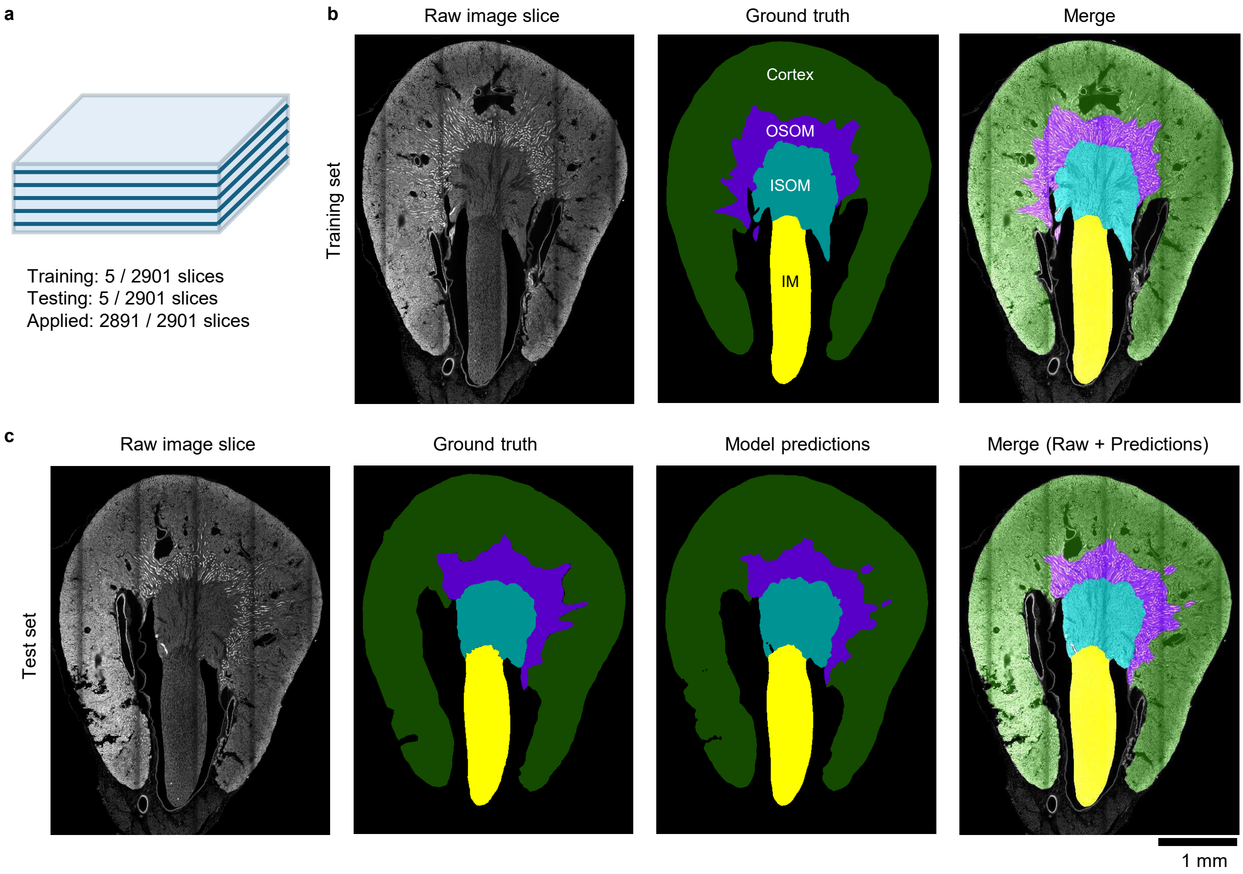

Supplementary Figure S 6: nnU-Net based segmentation of kidney tissue regions. An nnU-Net model, designed to handle diverse biomedical imaging datasets, was trained to segment four kidney tissue regions: cortex, outer stripe of outer medulla (OSOM), inner stripe of outer medulla (ISOM), and inner medulla (IM). (**a**) Ten slices spanning the whole volume were manually annotated: five slices (raw images and corresponding ground-truth masks) were used for training and five for testing. (**b**) An example slice (raw image, corresponding ground-truth masks, and merge) used for training is shown. The model achieved high intersection-over-union (IoU) scores across classes: (IM, 0.99; ISOM, 0.93; OSOM, 0.84; cortex, 0.96), with a mean IoU of 0.93. (**c**) An example slice from the test set comparing ground-truth masks and model predictions is also shown. The trained model was subsequently applied to the remaining slices to generate a 4-class segmentation of the entire volume.

Supplementary Figure S7

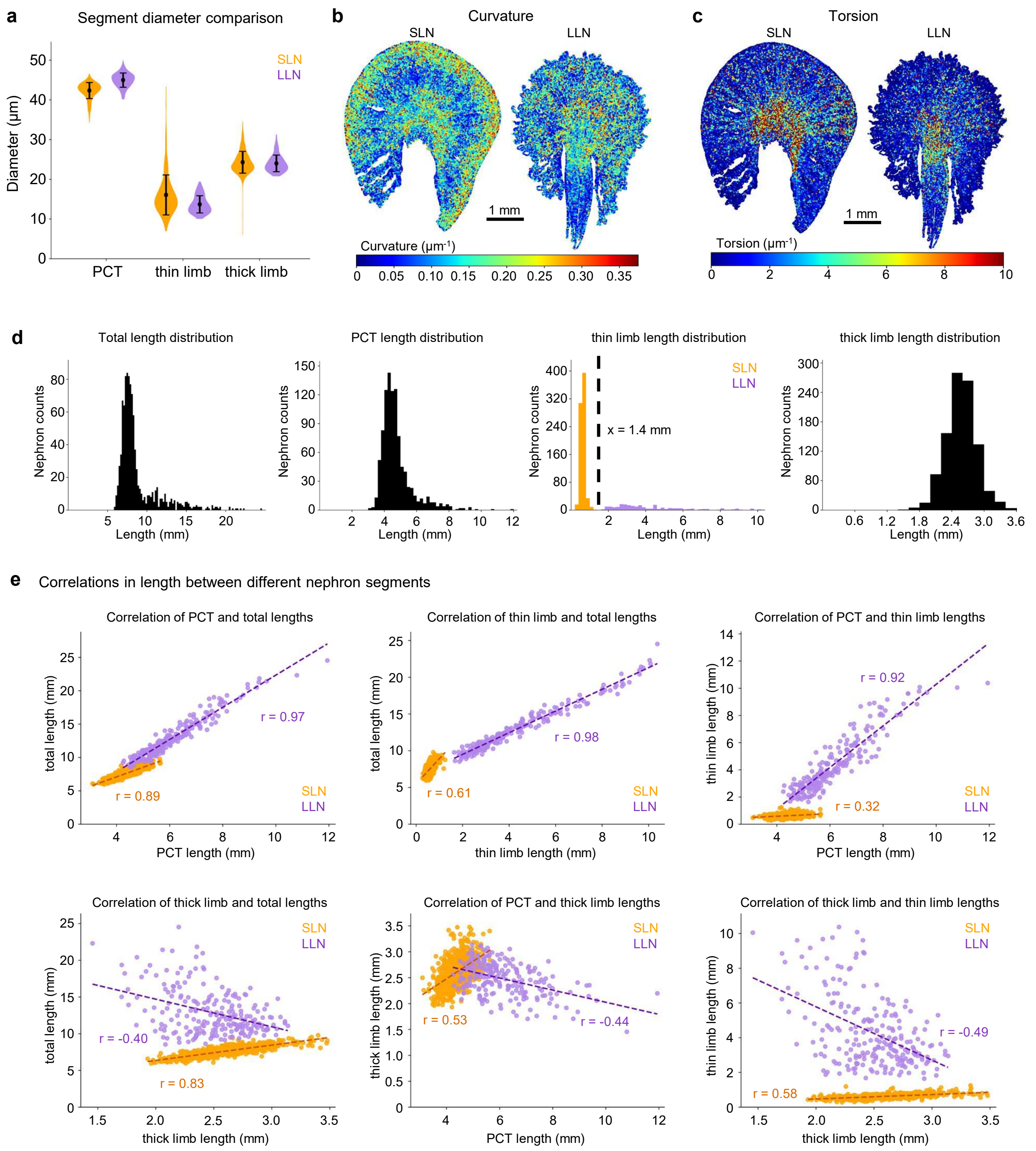

Supplementary Figure S7: Quantification of mesoscale organization of the kidney. (**a**) Violin plots of segment diameters for SLN and LLN nephrons (PCT, thin limb, and thick limb). Mean ± S.D. is shown. (**b-c**) Mesoscale distribution of curvature (b) and torsion (c) computed from all nephron traces. Values were downsampled by taking the maximum absolute value within each 20 × 20 μm^2 spatial bin in a 2D projection. (**d**) Histograms of total nephron length and segment-specific lengths (PCT, thin limb and thick limb) with a bin size of 0.2 mm. Thin limb lengths show a bimodal distribution and a length of 1.4 mm was used as the threshold to classify nephrons as SLN or LLN. (e) Pairwise correlation between lengths of different nephron segments and the total nephron length. r is Pearson correlation coefficient.

Supplementary Figure S8

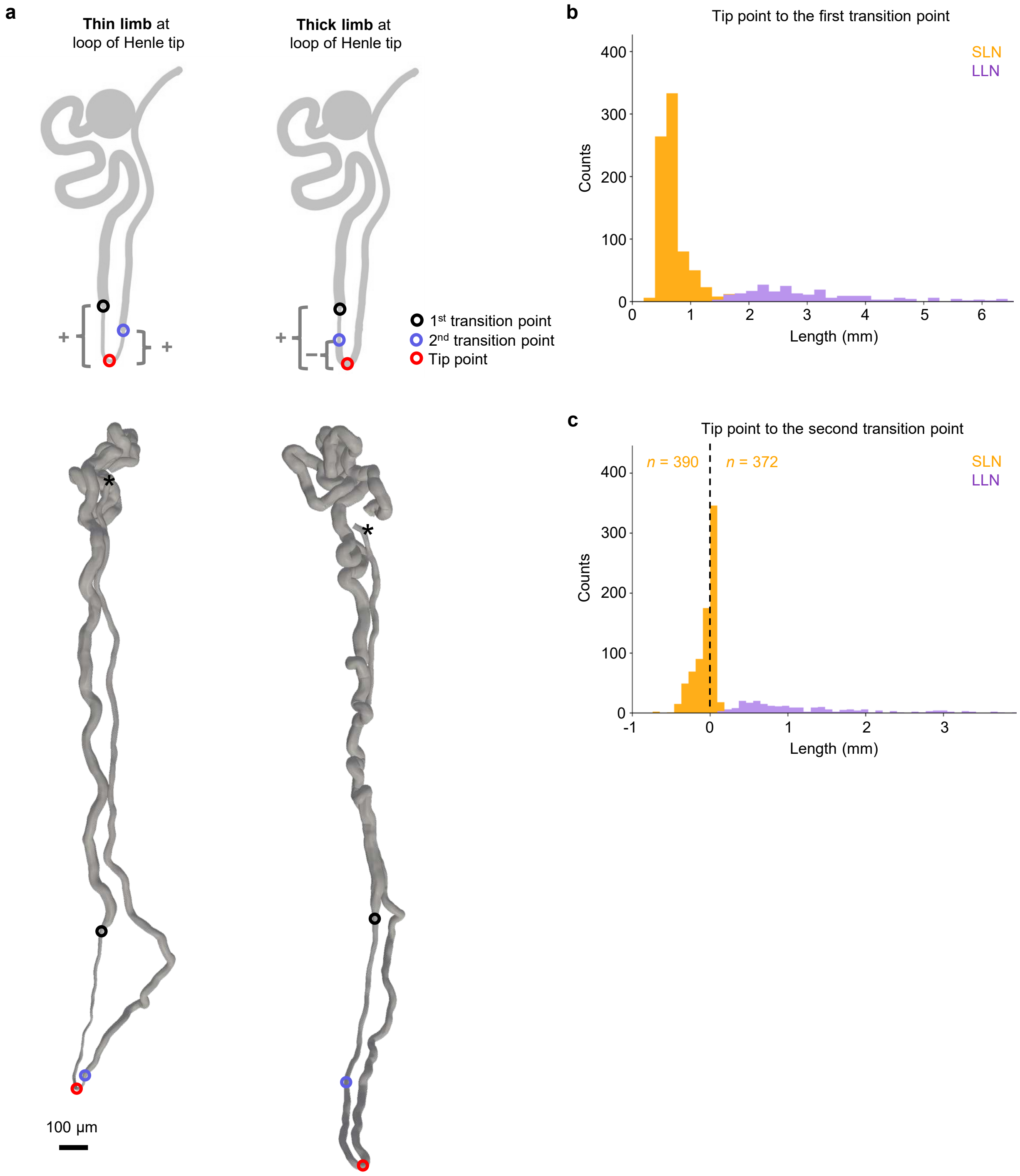

Supplementary Figure S8: Characterizing the presence of thin limb or thick limb at the loop of Henle tip. (**a**) Schematics and representative reconstructed nephron meshes showing either the thin limb or the thick limb at the loop of Henle. The first transition point corresponds to the PCT to thin limb transition. The second transition point corresponds to the thin limb to thick limb transition. The sign convention shown in (a) is applied in (b-c). (**b**) Histograms of distances from the tip point to the first transition point for SLN and LLN. The first transition is always before the tip point (all positive values) (**c**) Histograms of distances from the tip point to the second transition point for SLN and LLN. For LLN, all second transitions are after the tip point (all positive values). For SLN, the second transition point can be before or after the tip point (negative and positive values).

Supplementary Figure S9

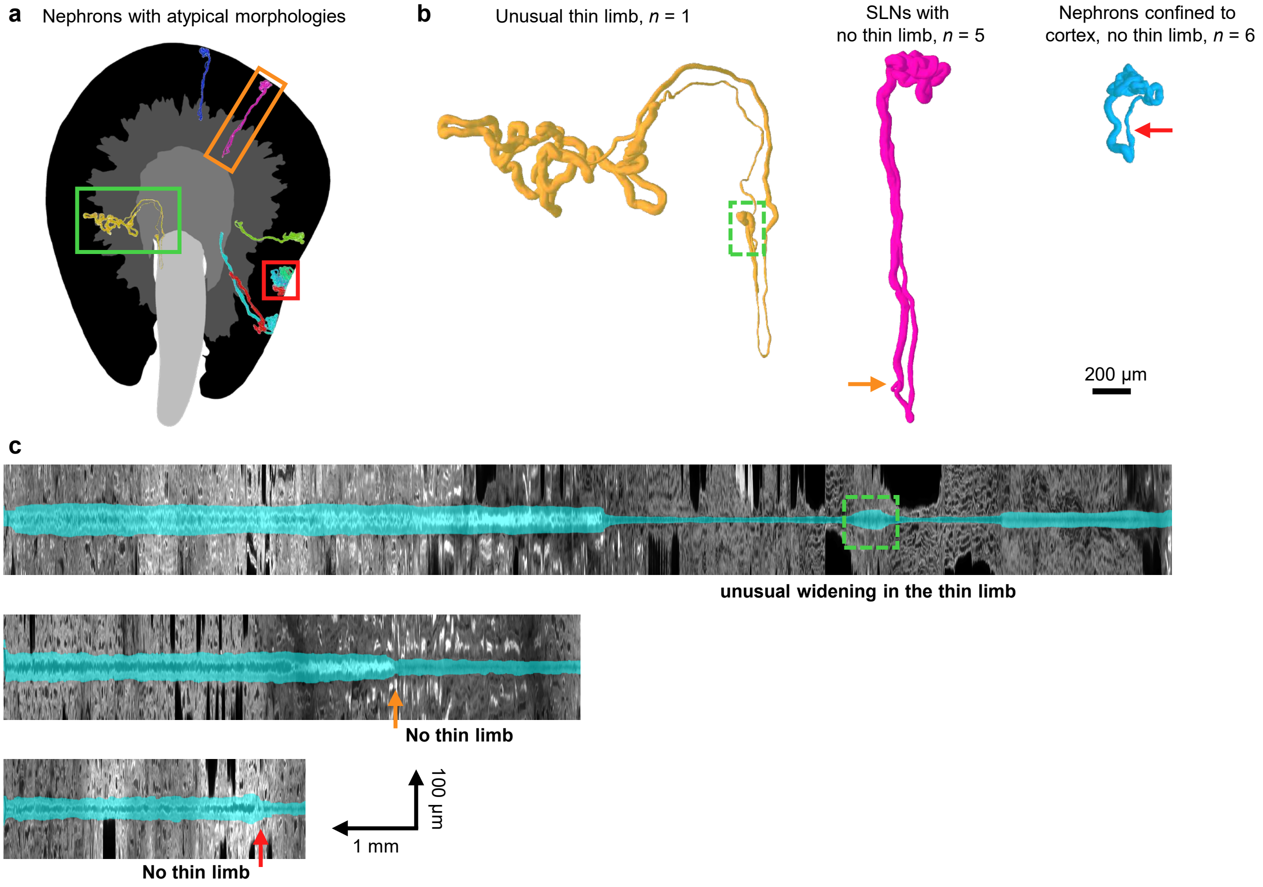

Supplementary Figure S9: Nephrons with atypical morphologies. (**a**) Spatial localization of nephrons showing atypical morphologies within corresponding tissue regions. Colored boxes mark the examples shown in (b). (**b**) Three representative mesh reconstructions illustrating the classes of atypical morphologies: a nephron with unusual widening within the thin limb (*n* = 1), SLN with no detectable thin limbs (*n* = 5) and nephrons confined to cortex without detectable thin limb (*n* = 6). (**c**) Representative straightened views with segmentation overlay. The green dashed box highlights an unusual widening that appears like the thick limb between thin limbs. Orange and red arrows indicate the transition point from PCT to thick limb.

Supplementary Figure S10

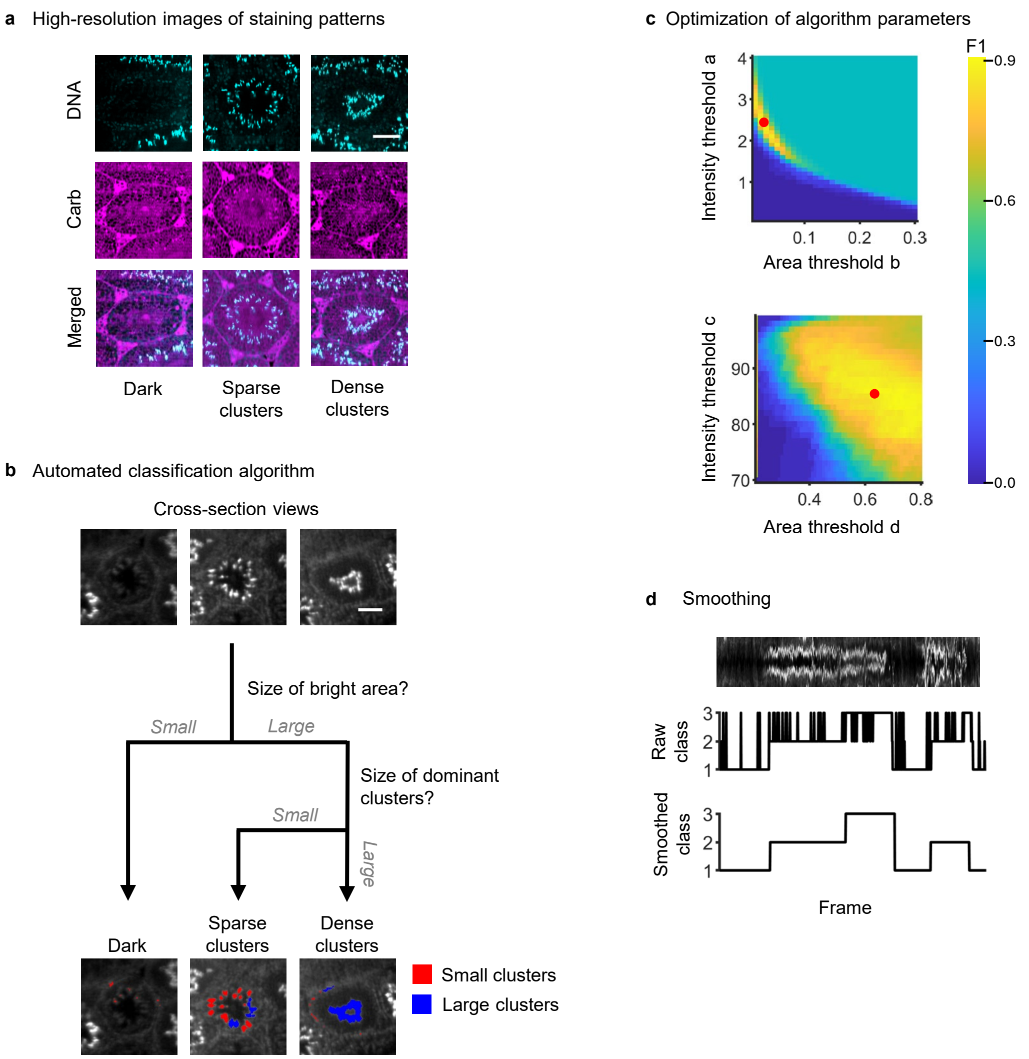

Supplementary Figure S 10: Analysis of spermatogenic waves in seminiferous tubules. (**a**) High-resolution images of carbohydrate and DNA channels exemplifying the three assigned classes: dark, sparse clusters, and dense clusters. (**b**) Schematic for hierarchical classification of seminiferous tubule cross-sections into the three classes using the DNA channel. (**c**) F1 score across the parameter optimization space. The optimal operating point is shown in red. All scale bars are 75 µm. (**d**) Straightened view of a seminiferous tubule, shown together with the raw classification and the smoothed classification obtained by a rolling mode filter.

Supplementary Figure S11: Screenshots of supplementary videos

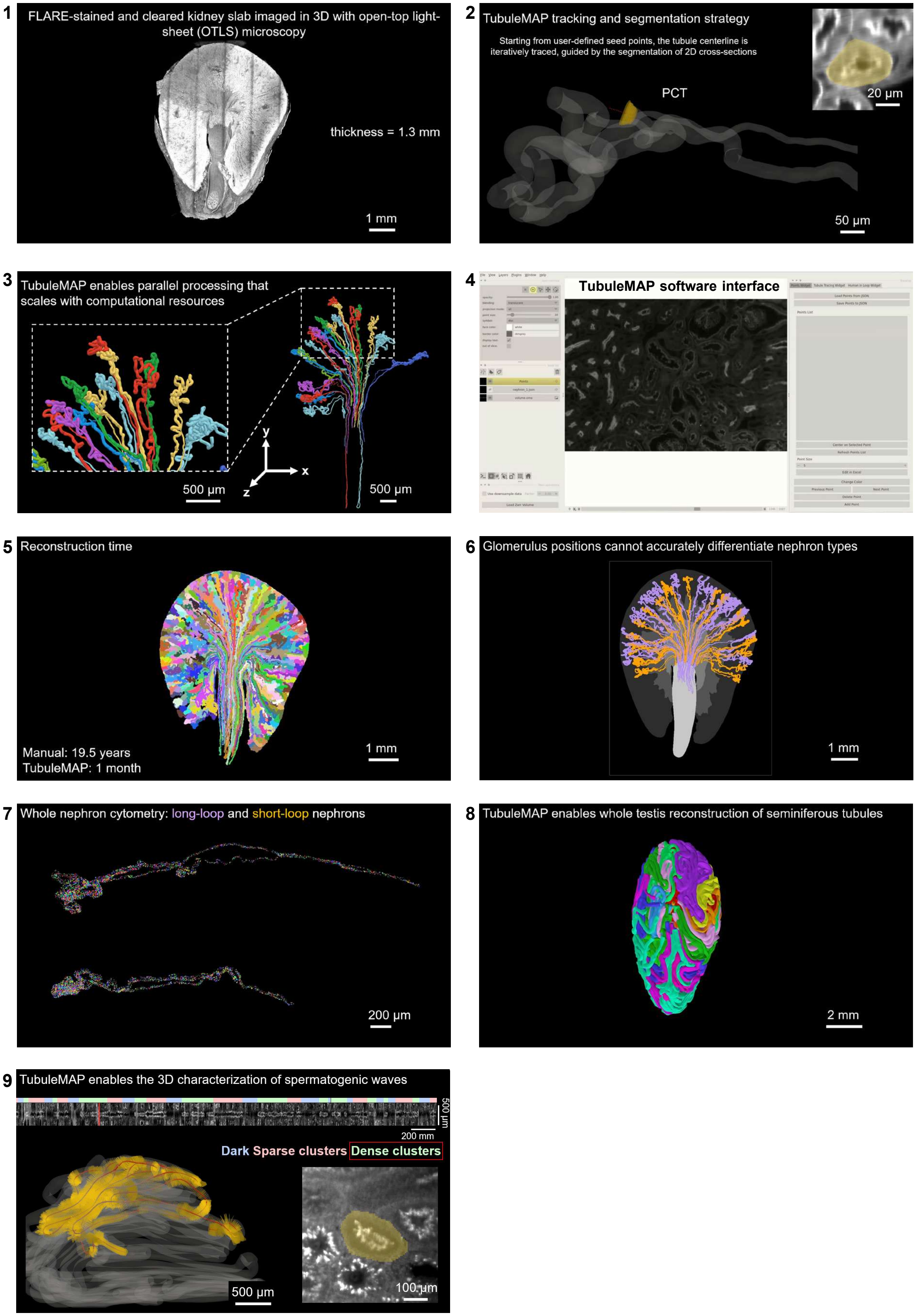

SUPPLEMENTARY VIDEOS

Supplementary Video 1: Surface rendering and virtual slices from kidney slab to cropped data with segmented nephron.

Supplementary Video 2: TubuleMAP tracking and segmentation strategy.

Supplementary Video 3: Parallel processing of tubules trajectories.

Supplementary Video 4: TubuleMAP graphical user interface and tracking workflow. Interface is based on napari with multiple widgets for data exploration and human intervention.

Supplementary Video 5: Three-dimensional reconstruction of 1000 nephrons from volumetric imaging data.

Supplementary Video 6: Spatial distribution of short-loop nephrons and long-loop nephrons across the kidney.

Supplementary Video 7: Nuclei distribution in short-loop and long-loop nephrons.

Supplementary Video 8: Three-dimensional visualization of seminiferous tubules in cleared testis tissue.

Supplementary Video 9: Top: straightened view of the tubule and assignment of spermatogenic wave states. Left bottom: Three-dimensional view of a mouse seminiferous tubule during tracking showing the centerline and orthogonal cross-sections with sampled planes (orange tiles). Right bottom: Raw image views and segmentation masks at the sampled orthogonal plane, along with classified spermatogenic wave states (dark, dense clusters, and sparse clusters).
